# Factor VIII Inhibitors in the Context of Renal Cell Carcinoma and Hemophilia A: A Tale of Two Causes

**DOI:** 10.7759/cureus.88651

**Published:** 2025-07-24

**Authors:** Rami Madani, Mohammad Z Khan, Adam Ayoub, Talal Al-Assil, Muhammad Usman

**Affiliations:** 1 Hematology and Medical Oncology, Western Michigan University Homer Stryker M.D. School of Medicine, Kalamazoo, USA; 2 Internal Medicine, California University of Science and Medicine, Colton, USA; 3 Hematology and Medical Oncology, Bronson Methodist Hospital, Kalamazoo, USA

**Keywords:** acquired hemophilia a (aha), autoantibodies, emicizumab, factor viii, renal cell carcinoma (rcc)

## Abstract

Hemophilia A (HA) is an X-linked recessive bleeding disorder caused by a deficiency of factor VIII (FVIII), leading to impaired secondary hemostasis. The cornerstone of HA management is replacement therapy; however, this can induce the formation of neutralizing inhibitors, resulting in a refractory FVIII deficiency that is unresponsive to recombinant therapy. This case report describes the postoperative development of FVIII inhibitors in a patient with mild HA and explores two likely etiologies contributing to inhibitor formation.

A man in his late 50s with a history of renal cell carcinoma (RCC) and mild HA (baseline FVIII: 22%; reference range: 55-200%) presented with right forearm and left lower leg swelling and pain two weeks after undergoing a radical nephrectomy. Preoperatively, he received 15 units/kg of recombinant FVIII, followed by 25-50 units/kg daily for six days postoperatively. After emergent causes were ruled out, laboratory evaluation revealed FVIII inhibitor levels of 9.1 Bethesda units (<0.5 BU) and FVIII activity <1%. The patient was started on monthly emicizumab therapy, resulting in resolution of inhibitor levels to <0.5 BU and improvement in FVIII levels to 6% within six months.

This case presents a diagnostic challenge in distinguishing the underlying mechanism of acquired hemophilia A (AHA). While inhibitor formation from recombinant FVIII therapy is well documented in severe HA, it is less common in mild cases with limited exposure. Alternatively, malignancy-associated immune dysregulation has been hypothesized to induce AHA, although the mechanism remains poorly understood. Given the rapid onset of inhibitor development following FVIII replacement and the absence of inhibitors during active RCC or resolution immediately after nephrectomy, FVIII exposure appears to be the more likely contributor in this case.

This case underscores the importance of careful monitoring for inhibitor formation even in patients with mild or previously asymptomatic HA, particularly in the perioperative setting. Clinicians should maintain a high index of suspicion for AHA in the appropriate clinical context, regardless of severity, to ensure timely diagnosis and management.

## Introduction

Hemophilia A (HA) is an X-linked recessive bleeding disorder caused by a deficiency of factor VIII (FVIII), leading to impaired secondary hemostasis. Common symptoms include prolonged bleeding, hemarthrosis, and excessive bleeding after injuries or surgeries. The cornerstone of HA management is replacement therapy, where exogenous FVIII is administered to replace the deficient protein [1]. 

The development of neutralizing inhibitors, antibodies that interfere with the function of the replacement clotting factor, following therapeutic infusion of FVIII is a significant complication in the treatment of HA. The cumulative incidence of inhibitors ranges from 20% to 40%; however, this metric is specific to severe HA as opposed to mild HA [2,3]. When these inhibitors develop, treatment with recombinant FVIII alone is ineffective. To detect and quantify these inhibitors, clinicians commonly use the Bethesda assay, a laboratory test that measures the level of FVIII inhibitors in a patient’s plasma and determines the inhibitor strength. 

Here, we report a case of acquired HA (AHA) presenting a diagnostic challenge, in which the underlying etiology was suspected to be either a primary malignancy, namely renal cell carcinoma (RCC), or a recent subacute blood transfusion. 

## Case presentation

**Figure 1:**
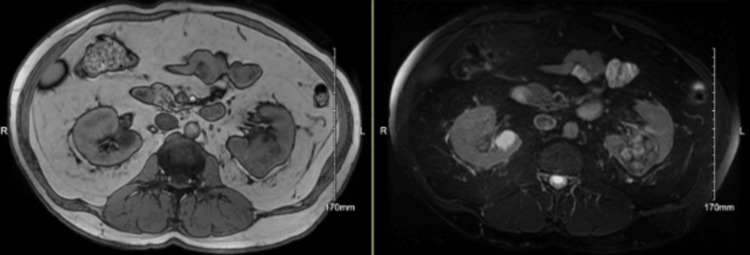
Axial slices from MRI of abdomen highlighting the multilobar renal mass in the left kidney. The left figure is a spoiled gradient recalled acquisition (SPGR) while the figure in the right is T2-weighted fat suppressed (T2FS).

A man in his late 50s with a history of RCC and mild HA with FVIII levels of 22% (reference range: 55-200%) presented with pain and swelling in his right forearm and left lower leg two weeks after radical nephrectomy. The patient had no history of kidney stones or genitourinary surgeries and has not had any carcinogen exposures, including a negative smoking history. His kidney function was preserved. RCC was diagnosed with MRI of the abdomen as shown in Figure 1, revealing a large, roughly 7 cm, lobulated, and heterogeneously enhancing mass in the left kidney. No tumor-related renal vein thrombus or evidence of metastatic disease was found, and the decision was for a radical left nephrectomy. 

The patient underwent robotic-assisted left radical nephrectomy, and given his history of HA, he was administered a total of 15 units/kg of recombinant FVIII before and throughout his procedure, in addition to 25-50 units/kg daily for six days postoperatively. Immunohistochemistry testing of the specimen confirmed Stage IIIa RCC of clear cell subtype. 

Despite treatment with antihemophilic factor, the patient experienced pain and swelling in two extremities (right forearm and left lower leg) two weeks post-operation. Deep venous thrombosis was ruled out with duplex ultrasound. On Bethesda assay, FVIII levels were found to be <1% (55-200%), and FVIII inhibitor was detected at 9.1 Bethesda units (<0.5 BU). Clotting studies were significant for an activated partial thromboplastin time (aPTT) of 121 s (25-39 s), leading to a diagnosis of AHA. The patient was initiated on emicizumab therapy and monthly monitoring thereafter. 

Six months post-diagnosis of AHA, the patient’s inhibitor levels were undetectable, with FVIII levels improving to 6%. The patient continues to be followed up with monthly monitoring of FVIII and FVIII inhibitor levels, as well as yearly abdominal MRIs and chest CTs. The patient remains in remission and symptom-free 3.5 years after diagnosis. 

## Discussion

While there exist multiple etiologies for bleeding associated with an underlying malignancy, commonly disseminated intravascular coagulation secondary to an adenocarcinoma, presentation and laboratory diagnostics can help distinguish AHA from other causes. Clinical manifestations include excessive spontaneous bleeding into the skin or soft tissues, often occurring with surgical operations. Although hemarthrosis is common to congenital HA, this complication is rarely seen in AHA [4]​​. Because AHA exclusively targets FVIII, patients will most often present with an isolated increase in aPTT, which specifically measures the intrinsic cascade of secondary hemostasis. From here, mixing studies, namely the Bethesda assay, are conducted. Diagnosis of AHA depends on evidence of neutralizing antibodies from the Bethesda assay along with decreased FVIII activity [1].​ 

Determining the trigger for AHA in our case is challenging, as both renal malignancy and FVIII transfusion are plausible contributors. Given this diagnostic complexity, it is important to explore both potential mechanisms for AHA development in this patient: immune dysregulation due to malignancy versus an immune response triggered by exogenous FVIII exposure. The following discussion will evaluate the plausibility of each mechanism, examining supporting and refuting factors to determine the most likely etiology. 

The association between hematologic neoplasms and solid tumors with AHA has been scarcely documented as a rare outcome resulting from malignancy [5].​​ In a review of 105 patients with AHA and associated solid tumors, prostate, lung, and colon cancers comprised nearly half of the malignancy-associated AHA cases [6].​​ The rarity of AHA and the paucity of research investigating the potential cross-reactivity of malignancy and FVIII have made it difficult to elucidate a causal mechanism for the development of AHA. However, it has been hypothesized that the tumor microenvironment contributes to micro-inflammation that favors increased activation of an adaptive immune response. One such marker of the adaptive immune response, B-cell activating factor (BAFF), has been found to be elevated in AHA patients. One research group found significantly higher levels of BAFF, a member of the tumor necrosis factor family, in patients with FVIII inhibitors compared to healthy controls and HA patients without inhibitors [7].​​ BAFF is known to be involved in the survival and maturation process of B-cells, and it plays a key role in the induction of humoral immunity; its overexpression contributes to the survival of autoreactive B-cells and thus destroys peripheral tolerance [8].​ Another study has established that the renal parenchyma expresses BAFF along with various other immune-related molecules, at baseline, and documented increased BAFF expression in patients with RCC, specifically the clear cell subtype (CC-RCC) [9].​ ​Additionally, the temporal relationship between the RCC diagnosis and AHA onset raises the possibility that tumor-related immune activation had been silently progressing before clinical detection, eventually culminating in overt FVIII inhibition that manifested after our patient's surgery. 

Alternatively, transfusion-related mechanisms leading to AHA involve a breakdown in immune tolerance to FVIII. While the development of inhibitors is rare in patients receiving FVIII replacement, studies suggest that it typically occurs after multiple exposures, with most cases developing within 10-15 days of cumulative infusions [1]. Certain risk factors, such as advanced age, underlying immune dysregulation, and genetic predisposition, may increase susceptibility. Exposure to exogenous FVIII, particularly in the context of transfusions or replacement therapy, can trigger an immune response in predisposed individuals. This response occurs through antigen presentation by activated dendritic cells, which internalize and process exogenous FVIII, presenting its epitopes on major histocompatibility complex (MHC) class II molecules to CD4+ T-helper cells. These T-helper cells activate B cells, leading to the production of high-affinity IgG antibodies that neutralize FVIII activity [10]. This immune activation may be exacerbated in patients with malignancies, as the tumor microenvironment often fosters systemic inflammation, immune dysregulation, and heightened antigen presentation [11]. In addition, surgical interventions and associated tissue damage can amplify immune responses through the release of pro-inflammatory cytokines and damage-associated molecular patterns, further increasing the likelihood of inhibitor development. As a result, patients with malignancy who undergo surgery may be at an even greater risk of developing inhibitors following FVIII exposure. 

In our patient, this mechanism is strongly supported by the temporality of events. Despite having mild HA with no prior bleeding complications, he developed significant inhibitor levels within seven days of FVIII exposure, leading to a marked reduction in clotting activity. This rapid inhibitor formation is particularly noteworthy given its rarity in mild HA, where inhibitors typically arise only after prolonged or repeated exposures [2,3]. Most patients with mild disease have missense mutations that allow for the continued production of structurally altered but partially functional endogenous FVIII. This endogenous expression fosters immune tolerance, making it uncommon for exogenous FVIII to be recognized as foreign [12]. In contrast, patients with severe HA often have null mutations leading to a complete absence of endogenous FVIII, predisposing them to inhibitor formation due to the immune system’s lack of prior exposure to the antigen [13]. The abrupt emergence of inhibitors following a limited number of FVIII infusions suggests a heightened immune response, potentially exacerbated by perioperative inflammation and immune system activation as a sequela of RCC. 

## Conclusions

This case highlights the diagnostic complexity of AHA in the setting of RCC and recent FVIII transfusion, emphasizing the need to consider multiple potential mechanisms. However, the rapid onset of inhibitor formation within a week of transfusion, rather than a gradual immune response over months or years, supports FVIII replacement therapy as a likely cause as opposed to RCC, within which we would expect inhibitor development during active malignancy and resolution post-nephrectomy, neither of which occurred. The disease progression of FVIII exposure and subsequent inhibitor formation, along with the known immune pathways involved in transfusion-related AHA, strongly favor this mechanism over malignancy-induced immune dysregulation. 

Given this case, it is important to recognize that inhibitor formation is possible even in patients with mild HA and a limited history of FVIII exposure or transfusions. Understanding the interplay between malignancy, transfusion, and immune activation is critical for optimizing perioperative management and mitigating the risk of AHA in high-risk patients. The timing of RCC diagnosis, anti-hemolytic therapy, and inhibitor development may offer insights into risk stratification and tailored therapeutic strategies for future cases. 

Further research is needed to elucidate the precise mechanisms underlying AHA in cancer patients, particularly in those with preexisting mild HA. Investigating tumor-associated antigens and donor-derived immune triggers could help identify predictive markers for inhibitor development, paving the way for targeted interventions to prevent or mitigate AHA in immunocompromised individuals with solid malignancies.
